# Oblongifolin C inhibits metastasis by up-regulating keratin 18 and tubulins

**DOI:** 10.1038/srep10293

**Published:** 2015-05-14

**Authors:** Xiaoyu Wang, Yuanzhi Lao, Naihan Xu, Zhichao Xi, Man Wu, Hua Wang, Xiyi Li, Hongsheng Tan, Menghong Sun, Hongxi Xu

**Affiliations:** 1 School of Pharmacy, Shanghai University of Traditional Chinese Medicine, Shanghai, 201203, P.R. China; 2 Engineering Research Center of Shanghai Colleges for TCM New Drug Discovery, Shanghai, 201203, P.R. China; 3 Key Lab in Healthy Science and Technology, Division of Life Science, Graduate School at Shenzhen, Tsinghua University, Shenzhen, 518055, P.R. China; 4 Stanley Ho Centre for Emerging Infectious Diseases, The Chinese University of Hong Kong, Hong Kong, P. R. China; 5 Department of Pathology, Fudan University Shanghai Cancer Center, Shanghai, 200032, P.R. China

## Abstract

Tumor metastasis is the main cause of cancer-related patient death. In this study, we performed a wound healing migration screen to search for a metastatic inhibitor within our library of natural compounds. We found that oblongifolin C (OC), a natural compound extracted from *Garcinia yunnanensis* Hu, is an effective inhibitor of metastasis in human esophageal squamous carcinoma Eca109 cells. The transwell migration and matrigel invasion assay results also showed that OC inhibits the migration of Eca109 cells and HepG2 cells. OC can increase the expression of tubulin, indicating that OC inhibits metastasis via tubulin aggregation. In addition, the Western blotting, real-time PCR, and immunostaining results indicated that OC increases the expression of keratin18. Furthermore, the knockdown of keratin 18 by small interfering RNAs inhibited the expression of tubulin and increased the metastasis of cancer cells, suggesting that keratin 18 is the upstream signal of tubulin and plays a vital role in metastasis. A subsequent study in a tail vein injection metastasis model showed that OC can significantly inhibit pulmonary metastasis, as revealed by immunohistochemistry staining. Taken together, our results suggest that OC inhibits metastasis through the induction of the expression of keratin 18 and may be useful in cancer therapy.

Esophageal cancer (EC) and hepatocellular carcinoma (HCC) are common lethal malignancy worldwide with the highest incidence in north central China. EC is divided into two histological types: adenocarcinoma and squamous cell carcinoma. Esophageal squamous cell carcinoma (ESCC) is the dominant histological type worldwide, particularly in China and other Asian countries^1^,^2^. Even though considerable advances in diagnosis, surgical techniques and chemoradiotherapy have been recently made, ESCC remains one of the most lethal cancers, and most patients die from its recurrence or metastasis^3^,^4^. HCC accounts for 90% of primary liver cancers and has a large amount of patients in China partially due to the high Occult hepatitis B infection rate^5^. The main treatment for HCC involves the surgical removal of tumors and liver transplantation. However, HCC is always associated with a risk for postoperative recurrence and metastasis^6^. Thus, there exists a need for further intensive research on ESCC and HCC to improve the patients’ quality of life and prolong survival time through the identification of new treatment approaches.

Metastasis is responsible for 90% of cancer patient deaths^7^. Cancer metastasis is a complex cascade that starts when a primary tumor forms and tumor cells break through the basement membrane (intravasation). These tumor cells then circulate through the blood, adhere to the capillary wall, escape from the blood vessel (extravasation), and proliferate to form metastasis. The key components of the metastatic process in biologically aggressive tumors include proliferation, migration, invasion and angiogenesis. Many research efforts have attempted to elucidate this metastatic process^8^,^9^, but the knowledge is quite limited due to the complexity of this process^10^. It is of critical importance to identify novel drugs for inhibiting tumor metastasis.

Natural products from plants continue to attract attention for the discovery of novel cancer chemopreventive agents^11^. *Garcinia* species have been studied for more than 70 years, and many bioactive compounds with anticancer potential have been identified. Xanthones, polycyclic polyprenylated acylphloroglucinols (PPAPs), and benzophenones are the main chemicals isolated from *Garcinia* plants^12^. Gambogic acid, a caged xanthone from *Garcinia hanburyi*, has been tested *in vitro* and *in vivo* as a novel anticancer agent that inhibits cell proliferation, angiogenesis, and metastasis^13^,^14^. From the last decade, we have collected all of the *Garcinia* plants in mainland China and used bioactivity-guided fractionation to obtain many active compounds^15^. We found that *Garcinia* species contained many special compounds, including xanthones, benzophenones, bioflavonoids, and biphenyls. Using different bioassay platforms, we were able to screen novel compounds targeting various signaling pathways. For instance, we have reported that oblongifolin C (OC), a PPAP purified from *G. yunnanensis* Hu, can activate the mitochondria-dependent apoptotic pathway by activating Bax translocation^16^. In a more recent study, we found that OC is an autophagic flux inhibitor that blocks autophagosome-lysosome fusion and autophagic degradation^17^^17^. To explore the diverse activities of natural compounds, it will be interesting to use multiple screening platforms to investigate their functions and detailed mechanisms.

In this study, we screened a library of natural compounds extracted from *Garcinia* species to identify novel metastatic inhibitors in ESCC and HCC through a wound healing migration assay. We report that OC exhibits potent metastatic inhibitory activity *in vitro* and *in vivo* through elevating the levels of keratin18 and tubulin. The knockdown of keratin 18 in Eca109 cells was found to partially reverse the effect of OC on metastasis, suggesting that keratin 18 plays an important role on ESCC metastasis. Notably, OC significantly prevents pulmonary metastasis in nude mice injected with ESCC cells via the tail vein without obvious potency. Our results suggest that screening for novel metastatic inhibitors from plants may be an efficient approach for the identification of lead compounds for anti-metastasis drug discovery.

## Results

### OC inhibits cell migration and invasion *in vitro*

**Figure 1:**
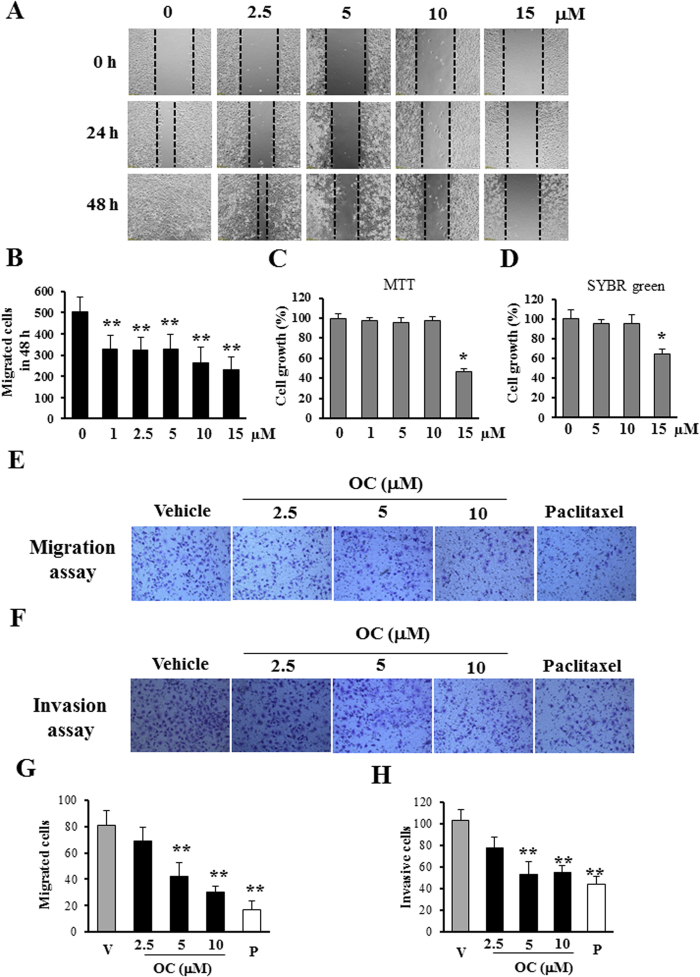
OC inhibits cell metastasis in Eca109 cells. (**A**) Wound healing assay. Eca109 cells were scraped, and the migration ability of the cells treated with or without different concentrations of OC was monitored with an inverted microscope. (**B**) The cell number from (A, 48 h) in the wounded regions was counted in each group from four independent experiments. (n = 4; **p < 0.01 vs. vehicle; Dunnett′s test). (**C**–**D**) Cell viability assays. MTT assay (**C**) and SYBR green assay (**D**) were applied to examine the cell viability in the absence or presence of different dose of OC for 24 h. (n = 8; *p < 0.05 vs. vehicle; Dunnett′s test). (**E**) Transwell assay. Eca109 cells were treated with different concentrations (2.5, 5 and 10 μM) of OC for 24 h, subjected to a transwell assay and detected through crystal violet staining. 100 nM paclitaxel was applied as positive control. (**F**) Matrigel invasion assay. Eca109 cell invasion was analyzed through a matrigel-coated transwell assay. The cells were treated with different concentrations (2.5, 5 and 10 μM) of OC for 36 h, and the invaded cells were stained with crystal violet. 100 nM paclitaxel was applied as positive control. (**G**–**H**) The cell number from transwell assay (**E**) and matrigel invasion assay (**F**) was counted in each group from four independent experiments. (n = 4; **p < 0.01 vs. vehicle; Dunnett′s test; V: vehicle; P: paclitaxel).

To identify novel metastasis inhibitors, we performed a functional screen using the wound healing assay with highly metastatic ESCC Eca109 cells^18^. We initiated the screen with PPAPs and xanthones extracted from *Garcinia* species^19^,^20^,^21^. Among all of the tested compounds, OC, which was extracted from *G. yunnanensis* Hu, exhibited preferential activity to inhibit cell migration. The chemical structure of OC is shown in Supplementary Fig. 1. As shown in Fig. 1A,B, and the Supplementary video, OC suppressed the numbers of migrated Eca109 cells in a concentration-dependent manner. We then compared the metastatic inhibition effects of OC and four commonly used anticancer drugs. Interestingly, 100 nM paclitaxel exhibited the same potent inhibition effect as 5 μM OC, 10 μM cisplatin exhibited less activation than OC, and etoposide (10 μM) and 5-fluorouracil (10 μM) did not inhibit cell migration in this metastasis screening platform (Supplementary Fig. 2 and unpublished data). To eliminate the possibility that the metastatic inhibition effect was due to cell proliferation inhibition, we examined the cytotoxicity of OC against Eca109 cells using MTT and SYBR green assay. As shown in Fig. 1C,D, 10 μM OC did not suppress cell growth for 24 h, suggesting that OC hardly inhibits metastasis by suppressing cell proliferation or inducing cell death at least in 24 h. Therefore, we selected the 10 μM for the mechanism study. To confirm the effect of OC on cell migration, we examined whether OC could inhibit migration in transwell assay. As shown in Fig. 1E,G, OC reduced the number of migrated cells in a dosage-dependent manner. Furthermore, as determined through a matrigel-coated transwell invasion assay, the number of invasive cells was also decreased after OC treatment (Figs. 1F,H). In addition, we tested the anti-migration effect of OC on other human cancer cell lines, including KYSE150 (EC cell line) and HepG2 (human liver carcinoma cell line). As shown in Supplementary Fig. 3A,B, OC suppressed the migration of KYSE150 cells in a dosage-dependent manner, as determined through wound healing assay. KYSE150 cell viability was also accessed by MTT assay in Supplementary Fig. 3C, indicating that OC did not affect cell proliferation in low concentration (<10 μM). In addition, we performed transwell assay (Supplementary Fig. 4A and 4B) and matrigel assay (Supplementary Fig. 4C and 4D) on KYSE150 cells. Consistently, OC exhibited strong inhibition activities in both assays. HepG2 cell line is one of the high metastatic HCC cell lines. The migration and invasion of HepG2 cells were also significantly prevented in the *in vitro* assays (Supplementary Fig. 5), indicating that OC could inhibit hepatocarcinoma metastasis. Altogether, our results showed that OC exerts strong effects on tumor cell migration and invasion in several cancer cell lines.

### OC increases the expression of keratin 18 in cancer cells

To investigate the detailed mechanism of OC against metastasis, we performed a 2D-PAGE and proteomics study to search for different expressed proteins as previous described^22^. The HepG2 cells were treated with OC for 24 h, and cell lysates were collected and subjected to 2D-PAGE in order to display their protein profiles. Supplementary Fig. 6A showed the representative pairs of silver-stained 2-DE images between two samples. Collectively, fifty-four protein spots were found to be differentially expressed (more than 3-folds changes) in the two pairs of samples. All the proteins were characterized by MALDI-TOF MS and MS/MS analysis and keratin 18 was found to be one of the most striking proteins raised by OC treatment (Supplementary Fig. 6B).

**Figure 2:**
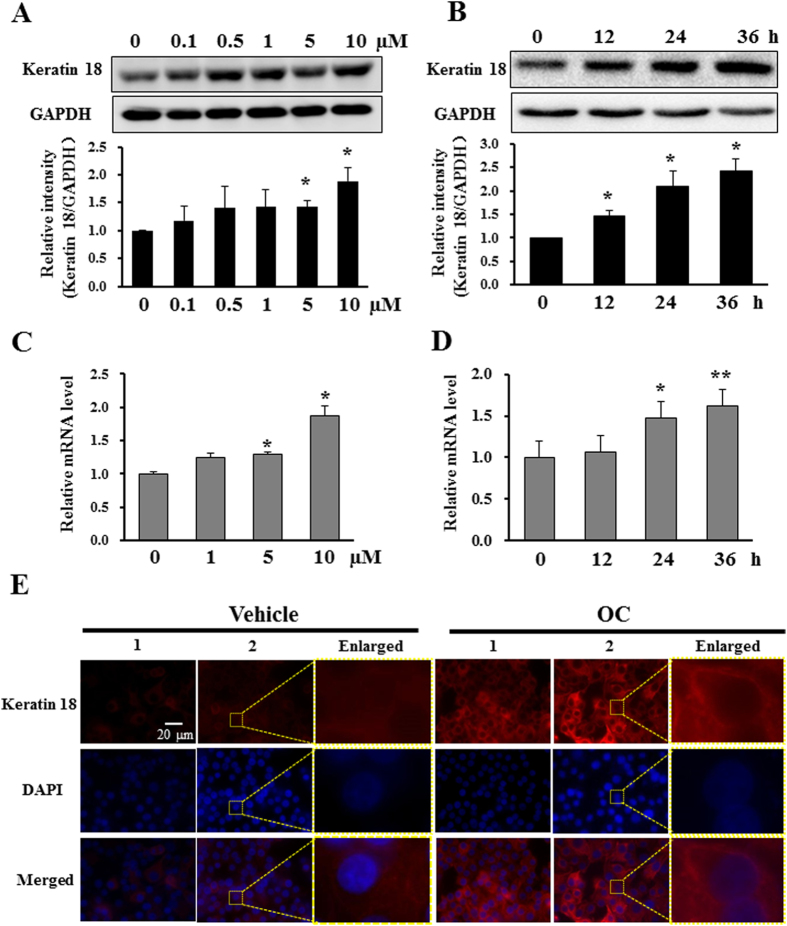
OC increases the level of keratin 18 in Eca109 cells. (**A**) Eca109 cells treated with different concentrations (0.1, 0.5, 1, 5 and 10 μM) of OC for 24 h were analyzed by Western blotting for keratin 18. GAPDH was used as a loading control. The statistic analysis from three independent experiments was shown in the lower panel. (*p < 0.05 vs. vehicle; Dunnett′s test). (**B**) Eca109 cells were treated with 10 μM OC over a certain time course, and the samples were analyzed by Western blotting for keratin 18 and GAPDH. The statistic analysis from three independent experiments was shown in the lower panel. (*p < 0.05 vs. vehicle; Dunnett′s test). (**C**) The relative keratin 18 mRNA levels (compared with GAPDH) were analyzed by quantitative real-time PCR. Eca109 cells treated with different concentrations (1, 5 and 10 μM) of OC for 24 h were analyzed by real-time PCR. (n = 3; *p < 0.05 vs. vehicle; Dunnett′s test). (**D**) Eca109 cells were treated with 10 μM OC over a certain time course, and the samples were analyzed by real-time PCR for keratin 18 and GAPDH (n = 3; *p < 0.05, **p < 0.01 vs. vehicle; Dunnett′s test). (**E**) Immunofluorescence analysis of Eca109 cells using an anti-keratin 18 antibody. The cells were treated with 10 μM OC for 24 h and detected with anti-keratin 18 antibody using a microscope. Scale bar = 20 μm. The enlarge images were shown in the right panel of each sample. All experiments were performed at least three independent times.

To confirm the proteomics results, Western blotting and real-time PCR assays were performed to examine the protein and mRNA changes of keratin 18 in OC-treated Eca109 cells. As shown in Fig. 2A,B, OC treatment elevated the expression of keratin 18 in a dosage- and time-dependent manner. In addition, the real-time PCR results also showed that OC can increase the mRNA levels of keratin 18 in a dosage- and time-dependent manner (Fig. 2C,D). We further examined the effect of OC on the distribution of keratin 18 through immunofluorescence assay. As shown in Fig. 2E, OC treatment increased the fluorescent intensity compared with vehicle treatment. In addition, OC did not induce any significant changes in cell shape and size. We then examined the expression of keratin 18 in HepG2 cells. As expected, the expression of keratin 18 was up-regulated by OC treatment (Supplementary Fig. 7A). The real-time PCR results also showed that OC can significantly increase the mRNA levels of keratin 18 in HepG2 cells (Supplementary Fig. 7B and 7C). In addition, we did not found obvious change on the other keratin family proteins including keratin 8 and keratin 19 upon OC treatment (Supplementary Fig. 8). Taken together, these results indicated the existence of an inverse correlation between the expression of keratin 18 and metastasis, suggesting that keratin 18 may play a pivotal role in cancer cell metastasis.

**Figure 3:**
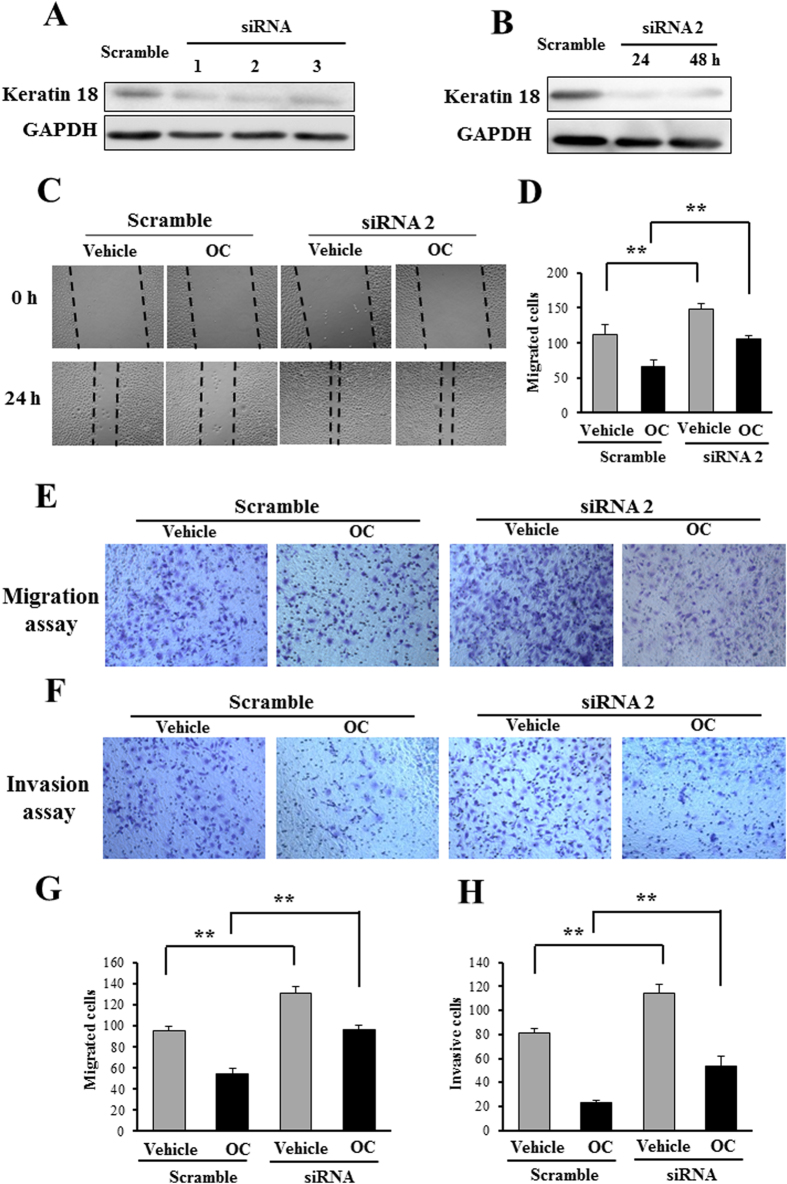
Keratin 18 knockdown partially eliminates the inhibitory effect of OC on metastasis. (**A**) Eca109 cells were transfected with scramble siRNA or individual siRNAs (No. 1 to 3) to keratin 18 for 24 h and analyzed by Western blotting. (**B**) Eca109 cells were transfected with keratin 18 siRNA (#2) for 24 and 48 h and analyzed by Western blotting. (**C**) Eca109 cells were transfected with keratin 18 siRNA (#2), and 24 h after transfection, the cells were treated with or without 10 μM OC and analyzed through a wound healing assay in 24 h. (**D**) The cell number from (**C**) in the wounded regions was counted in each group from four independent experiments. (n = 4; **p < 0.01 vs. vehicle; Student′s t-test). (**E**) Transwell assay. Eca109 cells were transfected with keratin 18 siRNA (#2), and 24 h after transfection, the cells were treated with or without 10 μM OC for 24 h, detected using a transwell assay and stained with crystal violet. (**F**) Matrigel invasion assay. Eca109 cells were transfected with keratin 18 siRNA (#2), and 24 h after transfection, the cells were treated with or without 10 μM OC for 36 h, detected using a matrigel invasion assay and stained with crystal violet. (**G** and **H**) The cell number from transwell assay (**E**) and matrigel invasion assay (**F**) was counted in each group from four independent experiments. (n = 4; **p < 0.01 vs. vehicle; Student′s t-test).

We then investigated whether the metastatic inhibition effect of OC was mediated by the increase of keratin 18. We silenced keratin 18 to examine whether the reduction of keratin 18 can abolish the inhibitory effect of OC on metastasis. As shown in Fig. 3A,B, all the three siRNAs exhibited strong efficacy for the knockdown of keratin 18. We then applied these siRNAs to further evaluate the effect of OC on metastasis. Firstly, we examined the migration ability of Eca109 cells after keratin 18 knockdown. As shown in Fig. 3C,D, the knockdown of keratin 18 by siRNA2 inhibited the migration of the cells, as determined through a wound healing assay. Importantly, the silencing of keratin 18 partially attenuated the effect of OC on migration, suggesting that keratin 18 plays a pivotal role on the regulation of migration. Secondly, we performed transwell and matrigel assays to further evaluate the effect of keratin 18 on migration and invasion. As shown in Fig. 3E,H, the transwell and matrigel assay results revealed that the silencing of keratin 18 efficiently enhanced Eca109 cell migration and invasion, and the effect of OC on metastasis was partially abolished by keratin 18 siRNA. Consistently, the other two siRNAs displayed similar effects of metastasis in the absence or the presence of OC (Supplementary Fig. 9). Taken together, our results indicated that keratin 18 is essential for the mediation of Eca109 cell metastasis and that OC may inhibit metastasis by up-regulating the expression of keratin 18.

### OC increases tubulin and inhibits ERK and AKT activation in Eca109 cells

**Figure 4:**
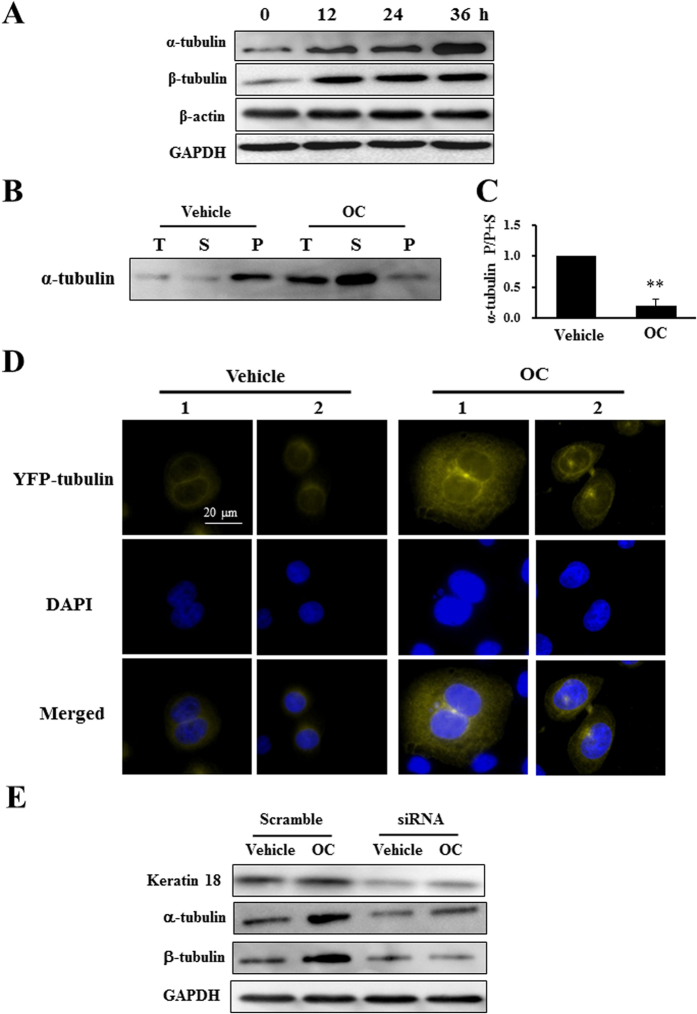
OC increases tubulin expression. (**A**) Eca109 cells treated with 10 μM OC for indicated time period were analyzed by Western blotting for α-tubulin, β-tubulin, β-actin and GAPDH. (**B**) Eca109 cells were treated with vehicle or 10 μM OC for 24 h. The total α-tubulin, soluble α-tubulin, and polymerized α-tubulin were separated and analyzed by Western blotting. (**C**) The statistic analysis of the proportion of polymerized and soluble α-tubulin from (**B**). (n = 3; **p < 0.01 vs. vehicle; Student′s t-test). (**D**) Eca109 cells were transfected with YFP-tubulin plasmid. After 24 h of incubation, the cells were treated with or without 10 μM OC for 24 h, and images were obtained using a microscope. Scale bar = 20 μm. (**E**) Keratin 18 suppression increases tubulin expression. Eca109 cells were transfected with keratin 18 siRNA (#2) for 24 h and treated with 10 μM OC for 24 h, and the samples were analyzed by Western blotting for keratin 18, α-tubulin, β-tubulin and GAPDH.

To further characterize the mechanism underlying the metastatic inhibition effect of OC and keratin 18, we continued to explore their influence on the cytoskeleton. The tubulin and actin families are key components of the cytoskeleton and play important roles in the cell metastasis. It has been suggested that keratin 18 mediates the disorganization of intermediate filaments in hepatocarcinoma cells^23^. We then examined the expression of α-tubulin, β-tubulin, and β-actin through a Western blot assay. OC (10 μM) increased the expression of α-tubulin and β-tubulin in a time-dependent manner, whereas β-actin was not altered by OC treatment (Fig. 4A). To examine if OC affects the proportion of soluble and polymerized microtubules, we applied hypertonic buffer to separate these two fractions of tubulin. As shown in Fig. 4B,C, 10 μM OC treatment for 24 h raised the soluble tubulin proportion. In addition, we overexpressed YFP-tubulin in Eca109 cells and acquired fluorescent images of Eca109 cells after OC treatment. The fluorescent intensity was significantly enhanced, whereas the cellular morphology and tubulin localization were unaffected (Fig. 4D). We then explored the relationship between keratin 18 and alterations in tubulin and found that both the α- and β-tubulin levels decreased after the knockdown of keratin 18. Importantly, the silencing of keratin 18 diminished the tubulin accumulation upon OC treatment, suggesting the keratin 18 was the key component that mediated the effect of OC on tubulins (Fig. 4E).

**Figure 5:**
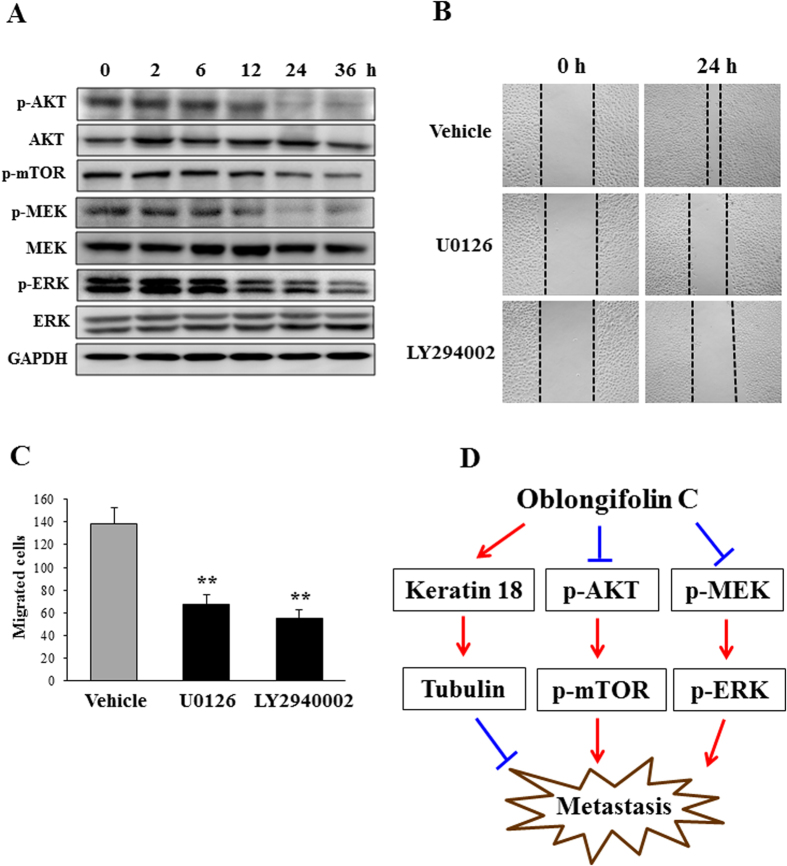
OC inhibits AKT and ERK activation. (**A**) Eca109 cells treated with 10 μM OC for indicated time period were analyzed by Western blotting for p-AKT, AKT, p-mTOR, p-MEK, MEK, p-ERK, ERK and GAPDH. (**B**) Eca109 cells were scraped, and the migration ability of the cells treated with U0126 (10 μM) and LY294002 (20 μM) was monitored with an inverted microscope. The images were acquired at 0 and 24 h. (**C**) The cell number from (**B**) in the wounded regions was counted in each group from three independent experiments. (n = 3; **p < 0.01 vs. vehicle; Student′s t-test). (**D**) Proposed model of OC-inhibited cell metastasis pathways. OC targets multiple signaling pathways including keratin 18/tubulin, AKT/mTOR, and MEK/ERK pathways to inhibit metastasis.

Weng *et al.* reported that keratin 18 acts as a target of AKT in the PI3K/AKT pathway and of ERK1/2 in the ERK/MAPK pathway^24^. In addition, Zhou *et al.* found that the ERK inhibitor U0126 and the AKT inhibitor LY294002 inhibit the expression of keratin 18 in epithelial ovarian cancer^25^. Furthermore, recent studies have implied the importance of the ERK pathway in cancer cell migration^26^ and demonstrated that LY294002 blocks breast cell migration^27^. In the above study, we showed that knockdown of keratin 18 could not totally eliminate the effect of OC on metastasis, suggesting that OC might target other signaling pathways. We then examined weather OC can affect the activation of ERK or AKT through a Western blot assay. As shown in Fig. 5A, OC suppressed AKT, mTOR, MEK and ERK phosphorylation compared with the vehicle, suggesting that OC suppresses Eca109 cell migration and invasion by down-regulating the AKT and ERK pathways. To further confirm that inhibition of AKT/mTOR and MEK/ERK contribute to esophageal cancer cell migration, we applied the ERK inhibitor U0126 and the AKT inhibitor LY294002 to Eca109 cells and measure their effect using wound healing assay. As shown in Fig. 5B,C, U0126 and LY294002 significantly suppressed Eca109 cell migration. Taken together, we hypothesized that OC targeted multiple signaling pathways including keratin 18/tubulin, AKT/mTOR, and MEK/ERK pathways to inhibit metastasis *in vitro* (Fig. 5D).

### OC inhibits pulmonary tumor metastasis in mice

**Figure 6:**
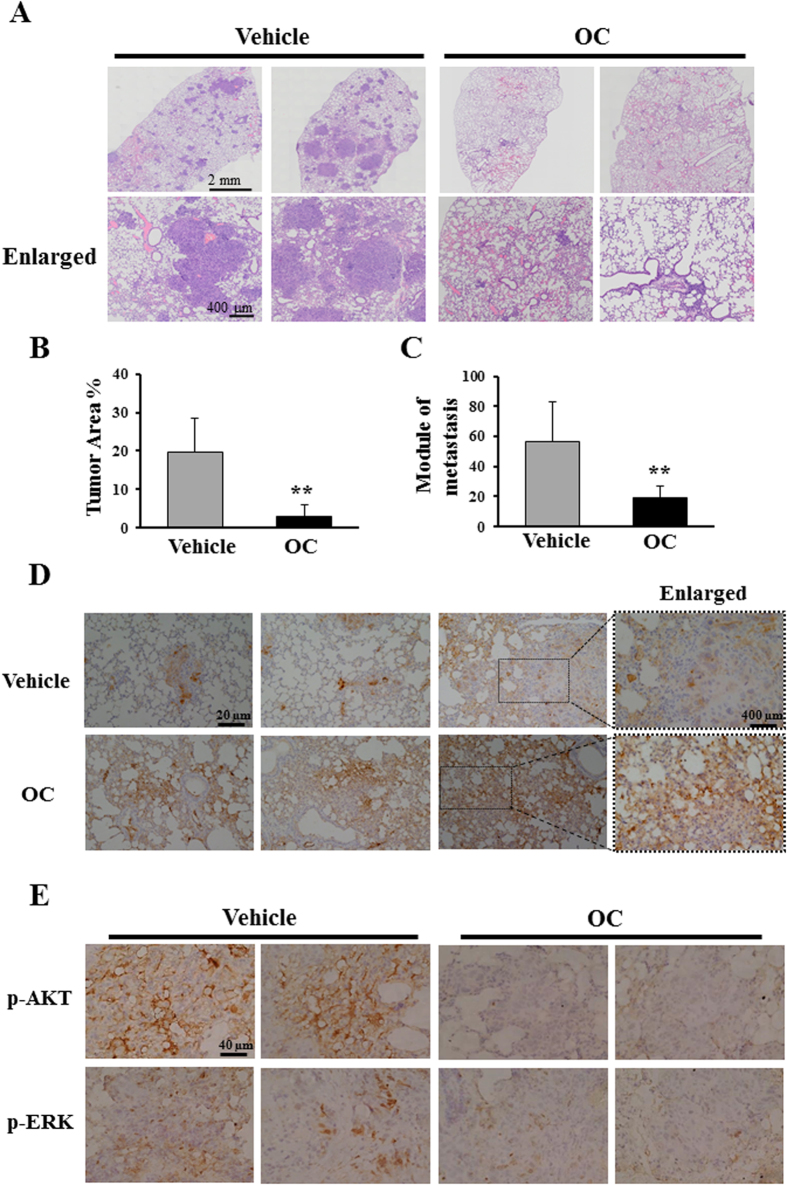
OC exhibits anti-metastasis effects in a murine experiment. (**A**) Five-week-old nude mice were injected via the i.v. route with KYSE150 cells and randomly divided into two groups. The tumor-bearing mice were then treated with vehicle or OC (30 mg/kg) via the i.p. route once every two days for a total of five weeks (n = 8). The histological analysis confirmed the presence of tumor lesions within the lungs. Representative examples of tumor lesions in the lungs from mice treated with vehicle or 30 mg/kg OC are shown. (**B**) Quantification of the effects of OC treatment on murine experimental metastasis. The tumor area from the mice was assessed using Olympus Soft Imaging Viewer (Japan, Tokyo) (n = 8; **p < 0.01 vs. vehicle; Student′s t-test). (**C**). The number of tumor modules was counted in all the mice (n = 8; **p < 0.01 vs. vehicle; Student′s t-test). (**D**) Immunohistochemical staining for keratin 18 in lung sections treated with vehicle or OC. (**E**) Immunohistochemical staining for phospho-AKT and phospho-ERK in lung sections treated with vehicle or OC.

To determine the anti-metastasis effect of OC *in vivo*, esophageal cancer KYSE150 cells were intravenously injected into nude mice via the tail vein, and the pulmonary metastasis was analyzed. A total of eight mice were randomly separated into two groups. Each of the two groups was injected once every two days through the i.p. route with either 30 mg/kg OC or saline as the control. OC was administered starting two days after the injection of the KYSE150 cells. Thirty-five days after tumor injection, the mice were sacrificed, and the pulmonary metastasis was examined by HE and immunohistochemistry staining. As shown in Fig. 6A, multiple metastatic foci with varying sizes were observed in the control mice, whereas the metastatic foci in the OC-treated mice were sparse and smaller. The statistic analysis using tumor area and tumor module counting indicated that OC significantly reduced the tumor area and module number in the lung tissues (Figs. 6B,C). Moreover, OC did not cause obvious side effects because no indications of weight loss were observed (Supplementary Fig. 10A). We then performed immunohistochemistry to detect the levels of keratin 18, phospho-AKT, and phospho-ERK. As shown in Fig. 6D, increased staining of keratin 18 in the lung tissue from OC-treated mice was o8bserved compared with the control mice, suggesting that OC may increase keratin 18 in the mice tumor model. The phospho-AKT and phospho-ERK were attenuated in OC-treated mice, which were consistent with *in vitro* observation (Fig. 6E). Other proteins including α-tubulin, β-tubulins, cleaved capspase-3 and TUNEL did not show obvious changes upon OC treatment *in vivo* (Supplementary Fig. 10B–D). Taken together, the results of our animal study indicated that OC elevates the expression of keratin 18, inhibits activation of AKT and ERK, and suppresses pulmonary metastasis of esophageal cancer cells without significant potency in mice.

## Discussion

Metastasis is the primary cause of lethality in cancer patients. For treatment of esophageal carcinoma and hepatocarcinoma, no effective anti-metastasis drug is currently available. We initiated this study with a wound healing assay to screen for active compounds targeting cell migration. Firstly, we found that OC is a potent inhibitor of metastasis in the wound healing screening platform. Secondly, we performed transwell and matrigel invasion assays to confirm the anti-migration and anti-invasion activity of OC. Notably, OC did not influence Eca109 cell proliferation under the conditions used in these assays. In addition, the wound healing assay showed that OC is more effective than clinical anticancer drugs, such as etoposide, 5-fluorouracil, and cisplatin (Supplementary Fig. 2). Thirdly, OC also inhibited hepatocarcinoma HepG2 cell metastasis *in vitro*, suggesting that OC exhibits a wide range of anti-metastasis effects against multiple cancer cells. Fourthly, we conducted an animal study and showed that OC has a strong inhibitory effect on pulmonary metastasis. Based on the above-described results, our research study indicates that the screening of novel metastatic inhibitors from natural products may be an efficient approach for the identification of novel lead compounds for cancer therapy.

A mechanistic study was conducted to investigate the effect of OC on esophageal cancer metastasis *in vitro* and *in vivo*. We first identified that OC inhibits tumor cell metastasis by up-regulating keratin 18 using proteomics analysis. Keratin 18 plays an important role in many cellular processes^24^. It has been reported that keratin 8/18 expression is abundantly found in invasive squamous cell carcinoma patients^28^ and that keratin 8/18 is a poor prognostic marker in squamous cell carcinoma of the esophagus and oral cavity^29^,^30^. Recent studies have indicated that keratin 8/18 loss can promote cancer cell migration^31^. In addition, Ha *et al.* found that the down-regulation of keratin 18 enhances the growth of breast tumor xenografts and invasiveness, suggesting that keratin 18 may refine the prognosis of breast cancer^32^. However, the detailed working mechanisms of keratin 18 on tumor metastasis remain unclear. Our study indicated that the knockdown of keratin 18 can enhance tumor cell migration and invasion in esophageal cancer. Consistent with our findings, a recent study reported that the staining for keratin 18 was weaker in human hepatocellular carcinoma (HCC) than in normal liver tissue^23^. Tubulin families are key components of the cytoskeleton and play important roles in cell metastasis^33^. Some anticancer agents, such as paclitaxel, a promoter of tubulin polymerization, have been found to exhibit clinical activity for metastasis therapy^34^. In our study, the silencing of keratin 18 reduced the expression of tubulin in Eca109 cells, suggesting that keratin 18 may be one of the upstream signals that regulate tubulins. Upon OC treatment, keratin 18-knockdowned cells maintain high metastasis and low tubulin levels. Thus, our results suggest that OC inhibits tumor metastasis partially by increasing the keratin 18 and tubulin levels.

However, as shown in Fig. 3 and Supplementary Fig. 9, knockdown of keratin 18 could not totally abolish the inhibitory effect of OC on metastasis, suggesting that OC might have other protein targets. We indeed found that OC inhibits AKT/mTOR and MEK/ERK phosphorylation, suggesting that OC suppresses metastasis via the AKT and ERK pathways in Eca109 cells (Fig. 5). Whilst, silencing keratin 18 did not alter the levels of AKT and ERK phosphorylation (data not shown), indicating that keratin 18 may be downstream of the AKT and ERK pathways. Zhou *et al* found that the AKT inhibitor LY294002 and the ERK inhibitor U0126 inhibited the expression of keratin 18 in epithelial ovarian cancer^25^. We also found LY294002 and U0126 significantly suppressed Eca109 cell migration, consistent with other reports^26^,^27^. However, the detailed mechanisms through which ERK and AKT mediate the elevation of keratin 18 and suppress metastasis still need to be explored.

In our animal study, OC significantly inhibited human esophageal cancer metastasis in the lungs. We previously reported that OC could inhibit the growth of MDA-MB-435 and HeLa cell-induced xenografts in nude mice^16^,^17^. In a more recent study, we demonstrated that OC suppresses cathepsin B expression in xenograft tissue *in vivo*^17^. Interestingly, cathepsin B also contributes to tumor metastasis in several types of cancers^35^,^36^. OC, as a natural compound, may act on multiple signaling pathways to exert its anticancer activities. Using different screening platforms, we found that OC effectively induces apoptosis, inhibits autophagic flux, and suppresses metastasis. These lines of evidence indicate that OC exhibits promising antitumor activity in various *in vivo* models. To further develop OC as a lead compound, it will be interesting and necessary to investigate the protein targets of OC.

## Materials and methods

### Reagents

RPMI 1640, Dulbecco’s modified Eagle’s medium (DMEM), penicillin, streptomycin, fetal bovine serum (FBS), BSA, Trizol reagent and Lipofectamine 2000 were purchased from Invitrogen (Carlsbad, CA, USA). PBS, MTT, DMSO, U0126, LY294002 and crystal violet were purchased from Sigma-Aldrich (St. Louis, MO, USA). Matrigel was purchased from BD (San Jose, CA, USA). The PrimeScript RT reagent kit was purchased from TaKaRa (Tokyo, Japan), and the SYBR Green Real-Time PCR kit was purchased from TOYOBO (Tokyo, Japan).

### Cell culture

Eca109 and HepG2 cells were purchased from the Shanghai Institute of Biochemistry and Cell Biology (Shanghai, China). KYSE150 cells were provided from Fudan University Shanghai Cancer Center. The Eca109 and KYSE150 cells were cultured in RPMI 1640, and the HepG2 cells were cultured in DMEM. All culture media were supplemented with 10% FBS, 100 U/ml penicillin and 100 mg/ml streptomycin (complete medium). The cells were maintained in a humidified atmosphere containing 5% CO_2_ at 37 °C.

### Wound healing migration assay

The cells were seeded on 24-well plates at a density of 1 × 10^5^ cells/well. A scrape was made through the confluent monolayer with a sterile plastic pipette tip. The plates were then washed twice with PBS and incubated in fresh complete medium at 37 °C in the presence or absence of the indicated concentrations of OC. The cells were maintained in a humidified atmosphere containing 5% CO_2_ at 37 °C using a live cell system (Tokaihit, Tokyo, Japan). The migrated distance of the cells was monitored and imaged under an Olympus microscope IX83 (Tokyo, Japan).

### Transwell assay

The cell migration was determined using a transwell chamber (Corning, Chelmsford St. Lowell, MA, USA) with a pore size of 8 μm. The cells were counted, and 5 × 10^4^ cells in FBS-free medium were placed in the upper chamber, whereas complete medium was added to the lower chamber. After incubation for 24 h at 37 °C, the cells in the upper chamber were carefully removed with a cotton swab, and the cells that had traversed to the reverse face of the membrane were fixed in methanol and stained with crystal violet. Five fields were selected randomly from the central and surrounding membranes and counted under a microscope.

### Matrigel invasion assay

The cell invasion was analyzed using a Matrigel-coated transwell with a pore size of 8 μm according to the manufacturer’s instructions. Briefly, 5 × 10^4^ cells were seeded in FBS-free medium in the upper chamber, and complete medium was added to the lower chamber. After incubation for 48 h at 37 °C, the cells in the upper chamber were carefully removed with a cotton swab, and the cells that had traversed to the reverse face of the membrane were fixed in methanol and stained with crystal violet. Five fields were selected randomly from the central and surrounding membranes and counted under a microscope.

### SYBR green assay

The SYBR assay was performed as previously described^37^. Briefly, the cells were treated with various concentrations of OC for 24 h. At the end of the incubation period, the medium was removed, and the cells were added to 100 μl of SYRB green (1:10,000) in lysis buffer (10 mM Tris-HCl pH 8, 5 mM EDTA, and 0.1% Triton X-100). After incubation for 30 min at room temperature, the fluorescence was determined using a Microplate Reader.

### MTT assay

The cells were treated with various concentrations of OC for 24 h. At the end of the incubation period, 10 μL MTT solution was added into each well of a 96-well plate for 4 h at 37^o^C, then 100 μL DMSO was added to dissolve the purple crystals. After shaking for 5 min, the optical densities at 595 nm were measured using a Microplate Reader.

### Plasmid construction and siRNA transfection

The plasmid encoding YFP-tubulin fusion proteins was kindly provided by Professor Donald C. Chang from Hong Kong University of Science and Technology. Keratin 18 siRNA (siRNA 1: 5’-GCUCAGAUCUUCGCAAAUATT-3’, 5’-UAUUUGCGAAGAUCUGAGCTT-3’; siRNA 2: 5’-GGUCAUUGAUGACACCAAUTT-3’, 5’-AUUGGUGUCAUCAAUGACCTT-3’; siRNA 3: 5’-GGACUUUAAUCUUGGUGAUTT-3’, 5’-AUCACCAAGAUUAAAGUCCTT-3’) and scramble control siRNA (5’-UUCUCCGAACGUGUCACGUdTdT-3’, 5’-ACGUGACACGUUCGGAGAAdTdT-3’) were purchased from GenePharma (Shanghai, China). The plasmid and siRNA fusion genes were transfected into Eca109 cells by Lipofectamine 2000 as instructed by the manufacturer. After 24 h of incubation, the cells were subjected to functional evaluations.

### Western blotting

The cells were lysed in ice-cold whole-cell extract buffer (50 mM Tris-HCl pH 8.0, 4 M urea, and 1% Triton X-100). The cell extracts were resolved by SDS-PAGE gel electrophoresis and transferred to a PVDF membrane. After blocking with 5% non-fat milk in Tris-buffered saline containing 0.2% Tween-20, the membranes were probed with the following antibodies: keratin 18 (Cat. 4548, Cell Signaling, MA, USA), p-AKT (Cat. 9271, Cell Signaling), AKT (Cat. 9272, Cell Signaling), p-ERK (Cat. 4695, Cell Signaling), ERK (Cat. 4370, Cell Signaling), p-MEK (Cat. 9154, Cell Signaling), MEK (Cat.9122, Cell Signaling), p-mTOR (Cat. 5536, Cell Signaling), β-tubulin (Cat. 5346, Cell Signaling), α-tubulin (Cat.sc-5286, Santa Cruz, CA, USA), keratin 8 (Cat. 53280, Abcam, Cambridge, UK), keratin 19 (Cat. 56625, Abcam) and GAPDH (Cat. 2251, Abcam). Following incubation with horseradish peroxidase-coupled secondary anti-mouse (Cat. 074-1806, KPL, Gaithersburg, MD, USA) or anti-rabbit antibodies (Cat. 474-1506, KPL), the protein bands were visualized using ECL Blotting Detection Reagents (Cat.54-61-00, KPL). Densities of the immunoreactive bands were evaluated using ATTO Densitograph Software Library CS analyzer (ATTO instruments, Tokyo, Japan).

### Detection of polymerized microtubules

The proportion of polymerized of microtubules was detected based on the protocol as previously described^38^. The Eca109 cells treated with OC or paclitaxel for 24 h before they were collected and washed. Then the cells were added with 200 μl hypotonic lysis buffer (MgCl_2_ 1 mM, EGTA 2 mM, 0.5% NP40, PMSF 2 mM, 10 μl protease inhibitor, Tris-HCl (pH 6.8) 20 mM) at 37 ^o^C in dark for 10 min. The supernatant and the precipitate were collected respectively. The supernatant containing soluble tubulin, while the precipitate was resuspended with 100 μl RIPA lysis buffer, this portion of the supernatant containing the original polymerized tubulin. Both the polymerized tubulin and soluble tubulin were detected at protein level, using Western blotting with the former.

### RNA isolation and quantitative RT-PCR

The total RNA isolation was performed using the TRIzol reagent following the protocol established by the manufacturer. Reverse transcriptional PCR was performed using the PrimeScript RT reagent kit. The qPCR analysis was performed in a Verti Thermal Cycler (Applied BioSystems) using the SYBR Green Real-Time PCR kit. The data collection was conducted using a StepOnePlus Real-Time PCR System Thermal Cycling Block (Applied Biosystems). The primers used for the qPCR reactions were as follows: keratin 18, 5’-GGCATCCAGAACGAGAAGGA-3’ and 5’-AGTGCTCCCGGATTTTGCT -3’; GAPDH, 5’-TGTTGCCATCAATGACCCCTT-3’ and 5’-CTCCACGACGTACTCAGCG-3’. The PCR reaction conditions were 10 s at 95 °C followed by 40 cycles of 5 s at 95 °C and 20 s at 60 °C.

### Immunofluorescence staining

The cells were grown on glass coverslips overnight, treated with or without OC for 24 h, fixed with 4% paraformaldehyde, washed with PBS for 5 or 10 min and then permeabilized using 0.3% Triton X-100 in PBS. After permeabilization, the cells were blocked with 5% BSA for 1 h and then incubated with keratin 18 antibody (diluted 1:100) overnight. The coverslips were washed with PBS and then incubated with Alexa-Fluor-594-conjugated anti-mouse-IgG secondary antibody (diluted 1:200; Invitrogen) for 1 h. The coverslips were then washed and mounted using DAPI, and images were obtained using an Olympus microscope.

### Murine experimental metastasis assay

The murine metastasis model was performed as previously described^10^. Briefly, four-week-old BALC/c male nude mice were purchased from the Experimental Animal Center of the Chinese Academy of Science (Shanghai, China) and maintained in a pathogen-free environment at the Experimental Animal Center in the Shanghai University of Traditional Chinese Medicine. All animal studies were conducted according to protocols approved by the Shanghai University of Traditional Chinese Medicine Animal Care and Use committee. The mice were intravenously injected with 1 × 10^6^ KYSE150 cells via the tail vein. After injection of the tumor cells, the mice were randomly dived into two groups and received an intraperitoneal injection of either saline or OC once every two days for five weeks.

### HE staining and immunohistochemistry

At the end of the treatment periods, the mice were sacrificed, and the lungs were immediately removed and fixed in 10% neutral buffered paraformaldehyde at 4 °C for 48 h. Selected samples were embedded in paraffin, sectioned and stained with hematoxylin and eosin (Sinopharm Chemical Reagent Co., Ltd.), keratin 18, p-AKT, p-ERK, α-tubulin, β-tubulin and cleaved caspase 3 (Cat. 8202, Cell Signaling). The primary antibodies were used at 1:500 for keratin 18, 1: 100 for p-AKT, 1: 100 for p-ERK, 1: 100 for α-tubulin, 1: 100 for β-tubulin, 1: 100 for cleaved caspase 3. The sections were finally mounted with DPX Mountant (Sigma, 317616) for histological analysis. Terminal deoxynucleotidyl transferase-mediated dUTP nick-end-labeling (TUNEL) assay was performed using a commercially available kit (Chemicon, Temecula, CA, USA) following the manufacturer’s instructions.

### Statistical analysis

All of the results are expressed as the means ±SD from three independent experiments. The statistical comparisons were performed using Student′s t-test and repeated-measures one-way ANOVA followed by post-hoc Dunnett′s test with the SPSS software (Version19, IBM, Somers, NY, USA). Values of *p* < 0.05 were considered significant.

## Author Contributions

X.W. wrote main protocol, carried out main experiments and statistical analyses, and prepared the manuscript. Y.L. designed the study and prepared the manuscript. N.X. designed the study, provided antibodies and prepared the manuscript. Z.X. wrote the protocol of SYBR green assay, and carried out part of wound healing migration assay. M. W. fed the mice, cultured the cells, and isolated the mRNA and protein. H.W. carried out 2-dimensional electrophoresis. X.L. and M. S. provided the KYSE 150 cells. H. T. provided OC. H.X. supervised the study and prepared the manuscript. All authors contributed to and have approved the final manuscript.

## Additional Information

**How to cite this article**: Wang, X. *et al.* Oblongifolin C inhibits metastasis by up-regulating keratin 18 and tubulins. *Sci. Rep.* **5**, 10293; doi: 10.1038/srep10293 (2015).

## Supplementary Material

Supplementary Information

Supplementary Movie 1

Supplementary Movie 2

Supplementary Movie 3

Supplementary Movie 4

Supplementary Movie 5
