# Supplementary material for: Oblongifolin C inhibits metastasis by up-regulating keratin 18 and tubulins
Source: Sci Rep. 2015 May 14;5:10293. doi: 10.1038/srep10293 (PMC4431421; doi:10.1038/srep10293)
Supplement: Supplementary Information [file srep10293-s1.pdf]

Oblongifolin C inhibits metastasis by up-regulating keratin 18 and tubulins

Xiaoyu Wang[1,2]*, Yuanzhi Lao[1,2]*, Naihan Xu[3]*, Zhichao Xi[1,2], Man Wu[1,2], Hua Wang[4], Xiyi Li[5], Hongsheng Tan[1,2], Menghong Sun[5#], and Hongxi Xu[1,2#]

[1] School of Pharmacy, Shanghai University of Traditional Chinese Medicine, Shanghai, 201203, P.R. China

[2] Engineering Research Center of Shanghai Colleges for TCM New Drug Discovery, Shanghai, 201203, P.R. China

[3] Key Lab in Healthy Science and Technology, Division of Life Science, Graduate School at Shenzhen, Tsinghua University, Shenzhen, 518055, P.R. China

[4] Stanley Ho Centre for Emerging Infectious Diseases, The Chinese University of Hong Kong, Hong Kong, P.R. China

[5] Department of Pathology, Fudan University Shanghai Cancer Center, Shanghai, 200032, P.R. China

Short title: Oblongifolin C inhibits cells metastasis

* **Authors contributed equally to this work**

Corresponding author:

Prof. Hongxi Xu, School of Pharmacy, Shanghai University of Traditional Chinese Medicine, Shanghai, 201203, China, Tel: 86-21-51323089, Email:xuhongxi88@gmail.com

Prof. Menghong Sun, Department of Pathology, Fudan University Shanghai Cancer Center, Shanghai, 200032, PR China, Email: menghongs@hotmail.com

Supplementary methods

Two-dimensional electrophoresis (2-DE), protein visualization and image analysis

OC or DMSO treated HepG2 cells were harvested and 2-DE was performed as described previously [1]. In brief, isoelectric focusing (IEF) was performed by using IPG strips (13 cm, pH 3–10). The samples containing 150 μg protein were diluted in a 250 μl rehydration buffer, and was subsequently loaded into IPG strips and rehydrated for 10 hr at 30 V with IPGphor II apparatus (Amersham Bioscience，Arlington Heights, IL, USA). Then IEF was carried out by following a stepwise voltage increase manner: 500 and 1,000 V for 1 hr and then 8,000 V for 6 hr. After the completion of IEF, the IPG strips were incubated in a balance buffer supplemented with 1 % DTT for 15 min under shaking. Subsequently, they were transferred to the balance buffer containing 2.5% iodoacetamide for another 15 min; the strips were loaded onto the top of a 12.5% uniform polyacrylamide gel slab for the two dimensional protein separation which was performed by using a 15 mA constant current for 30 min and then by using the 30 mA current. The procedures following 2-DE were performed as described in [1]. In brief, the gels were visualized by silver staining. These raw images were captured by using a GS-800 scanner (BioRad, CA, USA) and QuantityOne program (BioRad); the images were subsequently analyzed by the PDQuest program (version 8.0, BioRad). The differently expressed proteins spots were manually excised from the 2-DE gels. The gel

chips were incubated in a mixture of potassium ferricyanide and sodium thiosulfate for 5 min. After washing twice, the gel chips were equilibrated in ammonium bicarbonate and CAN with variant concentration and followed by vacuum-drying. Lastly, the gel chips were rehydrated with 25 mM ammonium bicarbonate (pH8.0) containing 10 mg/ml of trypsin at 37°C for 16 hr. After trypsinization, the peptide supernatants were spotted onto a target plate with CHCA as the matrix (4 mg/ml in 35 % ACN and 1 % TFA). The peptide samples were collected for mass spectrometric (MS) analysis by using a 4700 Proteomics Analyzer (TOF/TOFTM) (Applied Biosystems, CA, USA). A peptide mass mapping was performed by using a MASCOT program (Matrix Science, London, UK) against Swiss-Prot database with a GPS explorer software (Applied Biosystems).

Supplementary Reference

1. Fu, W.M. *et al*. Apoptosis induced by 1,3,6,7-tetrahydroxyxanthone in Hepatocellular carcinoma and proteomic analysis. *Apoptosis* **17**, 842-851(2012).

Supplementary Figure 1

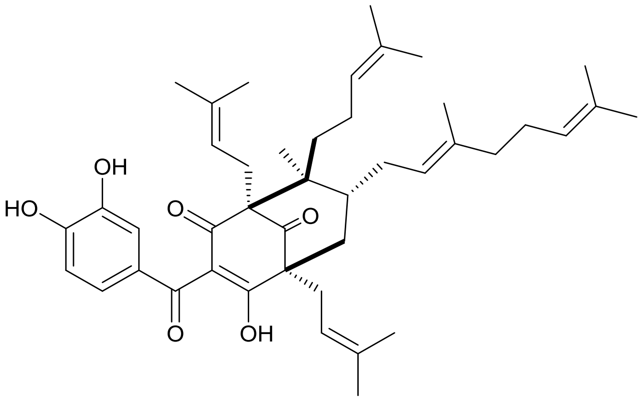

Oblongifolin C

Supplementary Figure 2

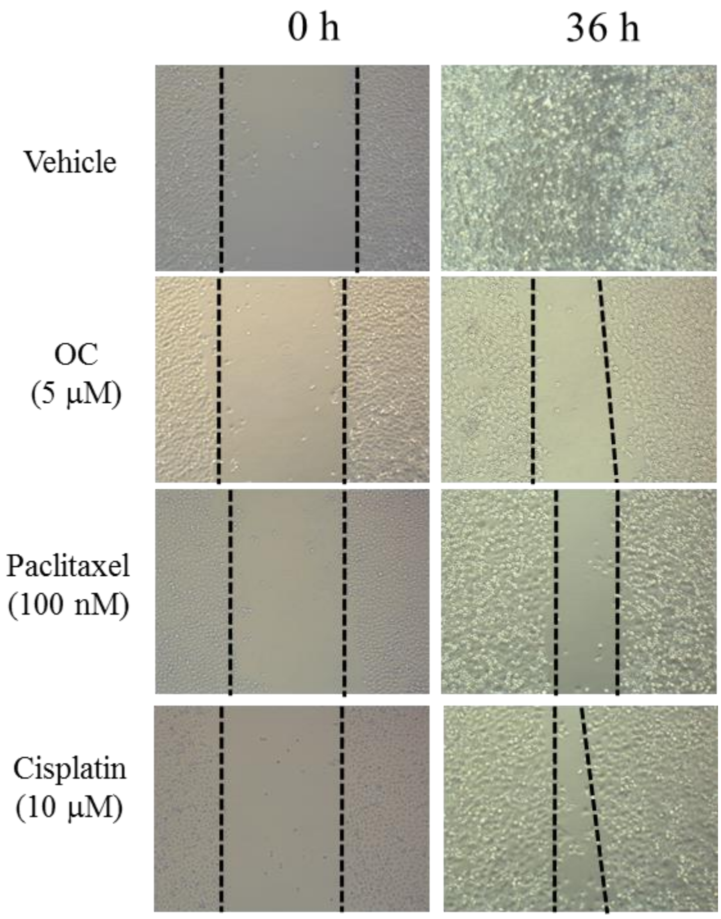

Supplementary Figure 3

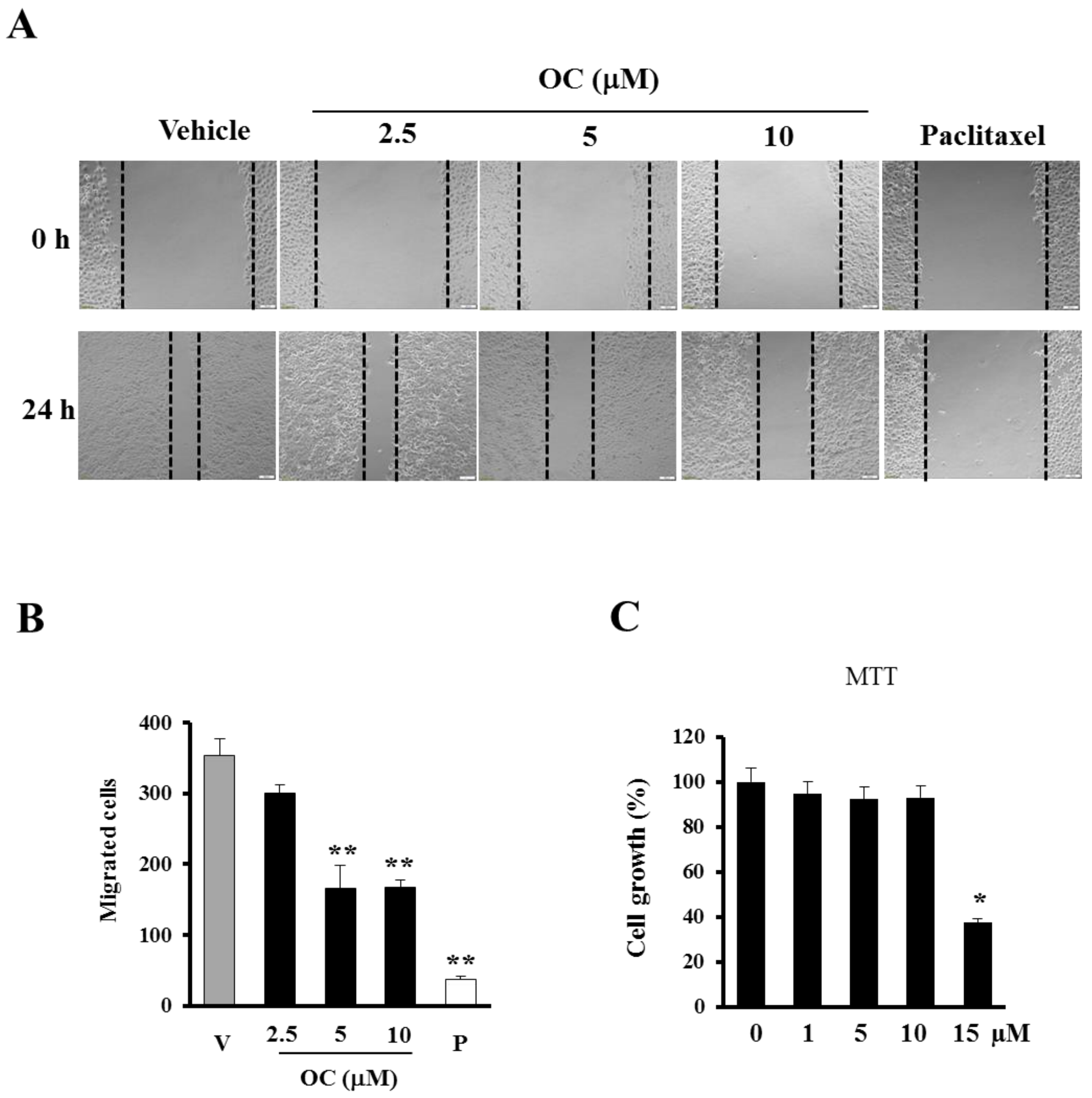

Supplementary Figure 4

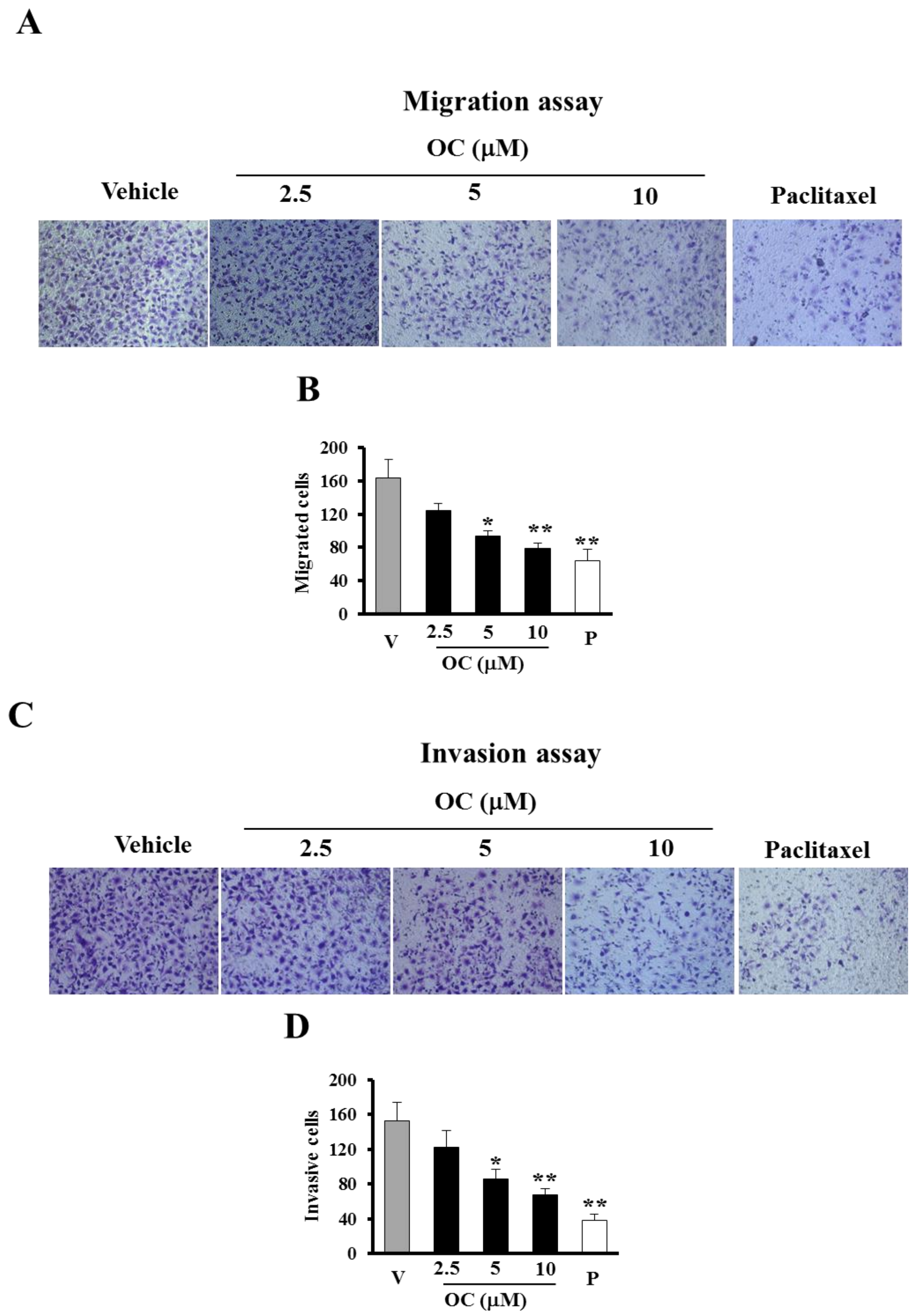

Supplementary Figure 5

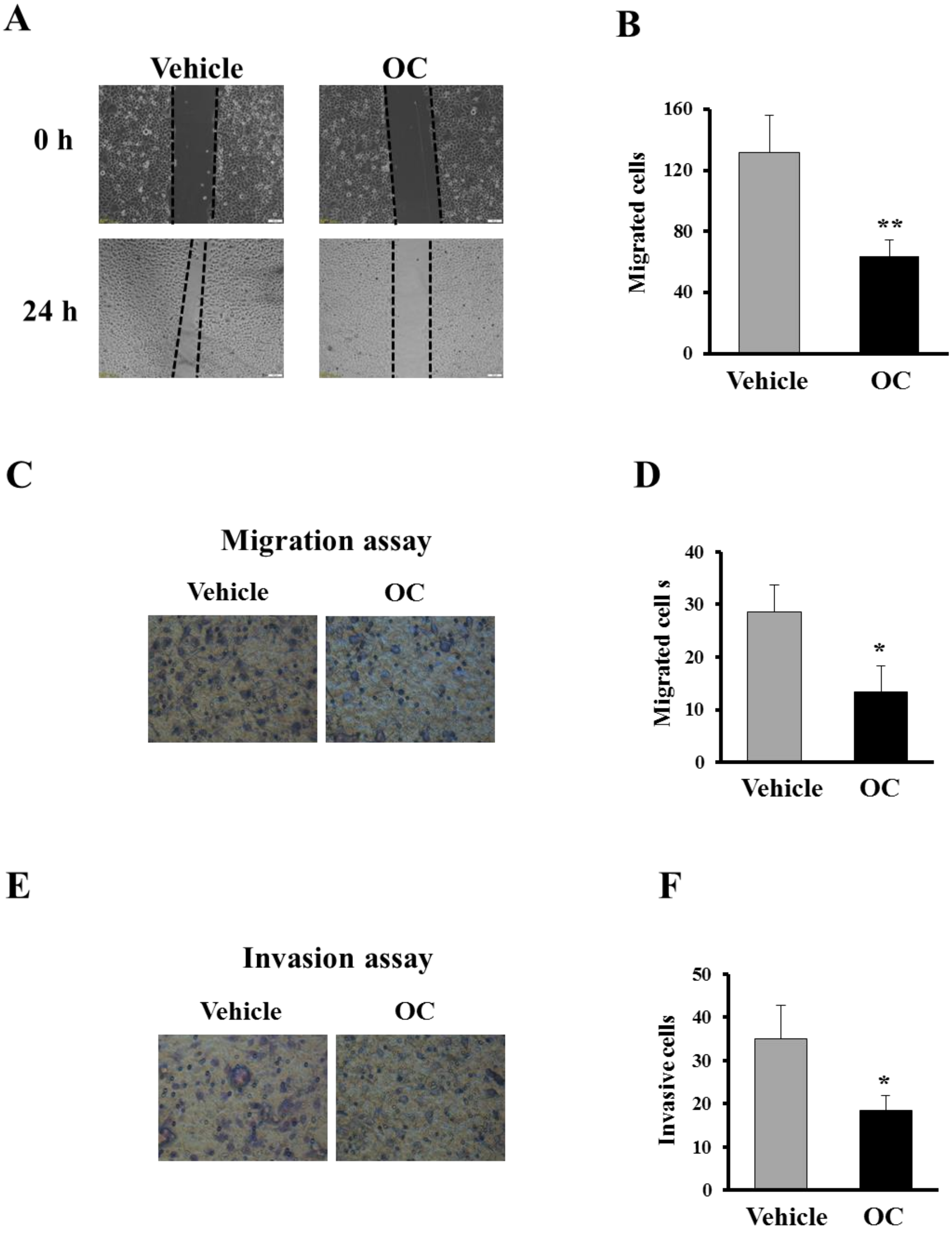

Supplementary Figure 6

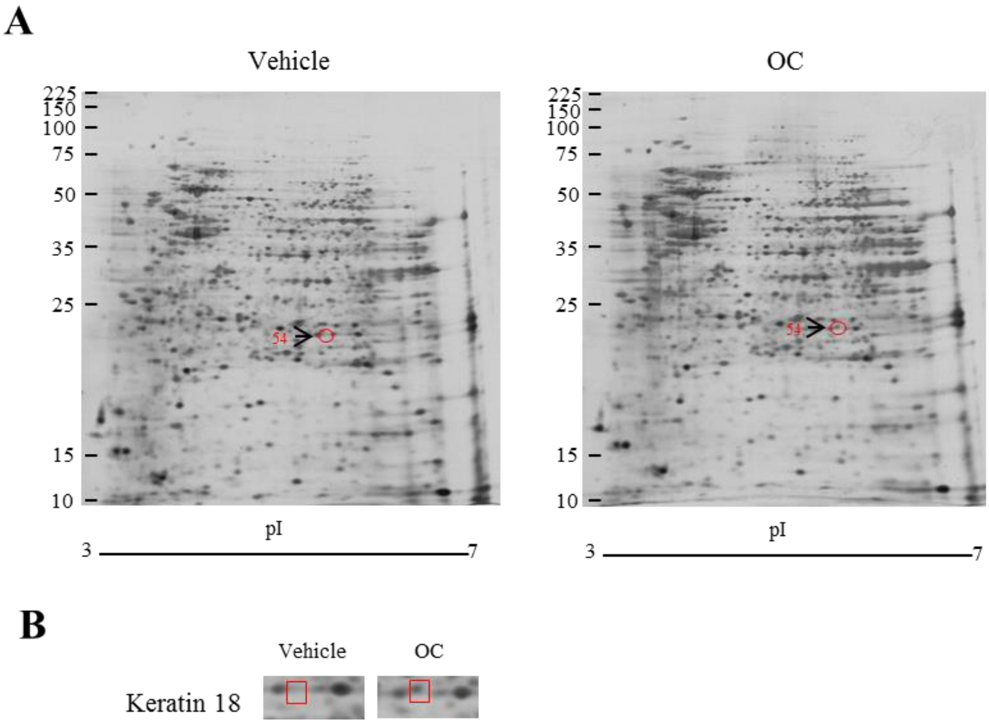

Supplementary Figure 7

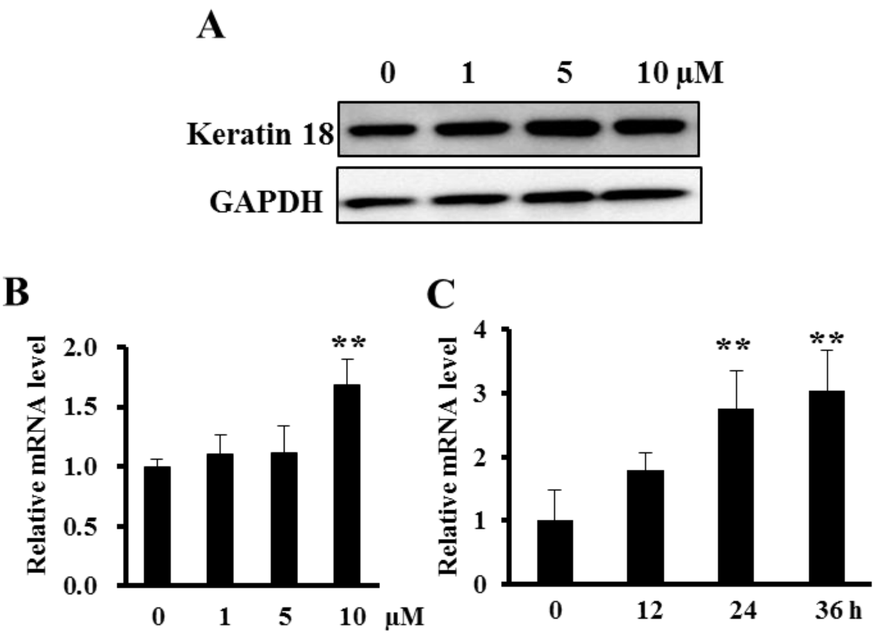

Supplementary Figure 8

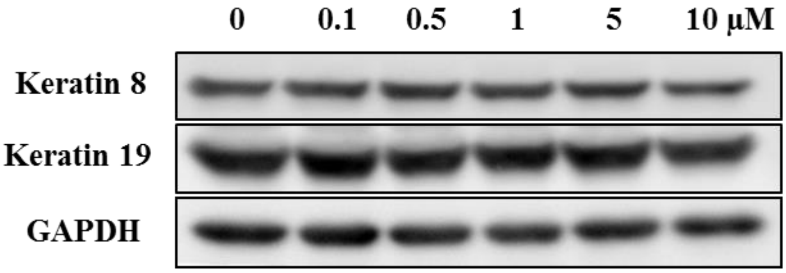

Supplementary Figure 9

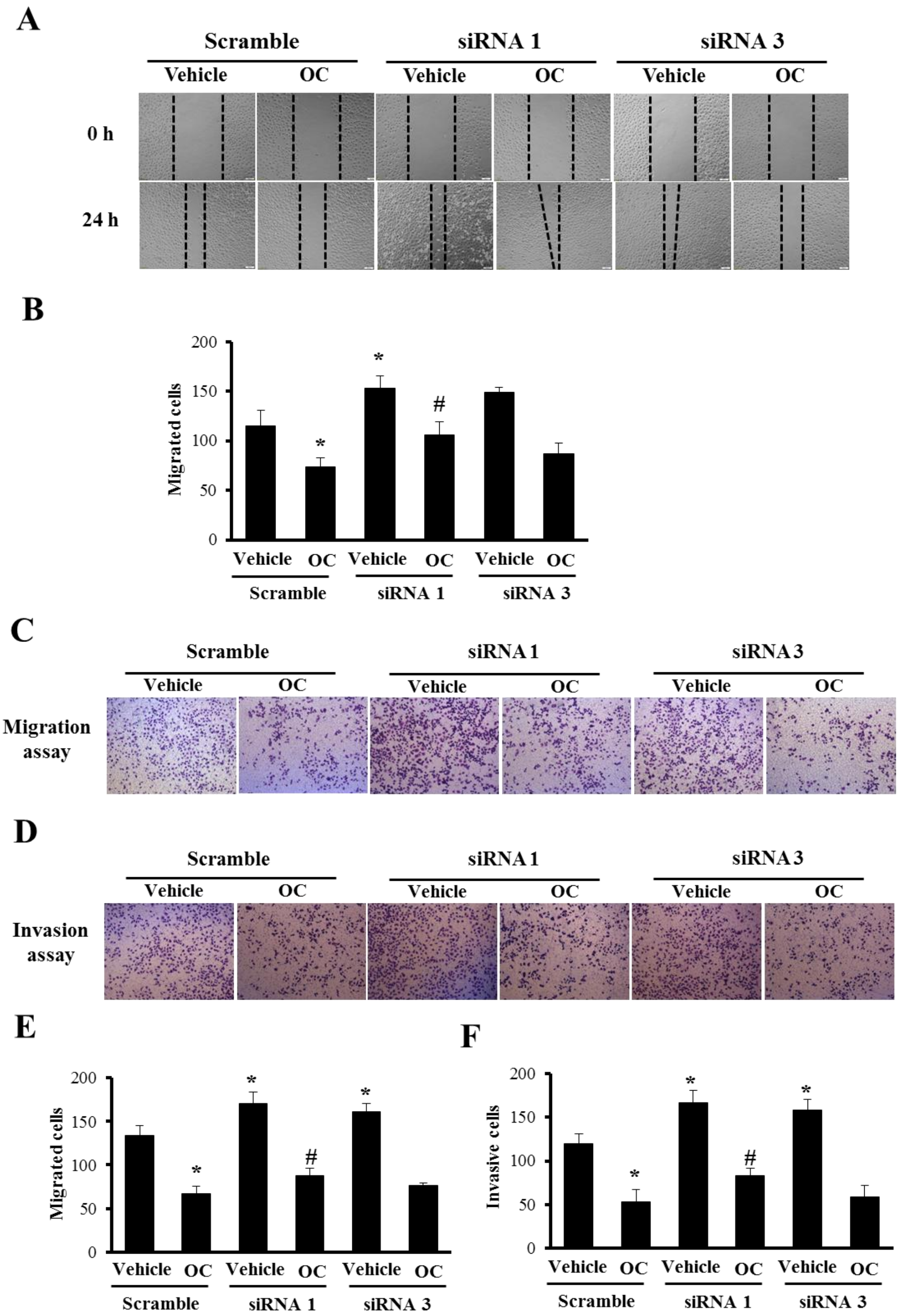

Supplementary Figure 10

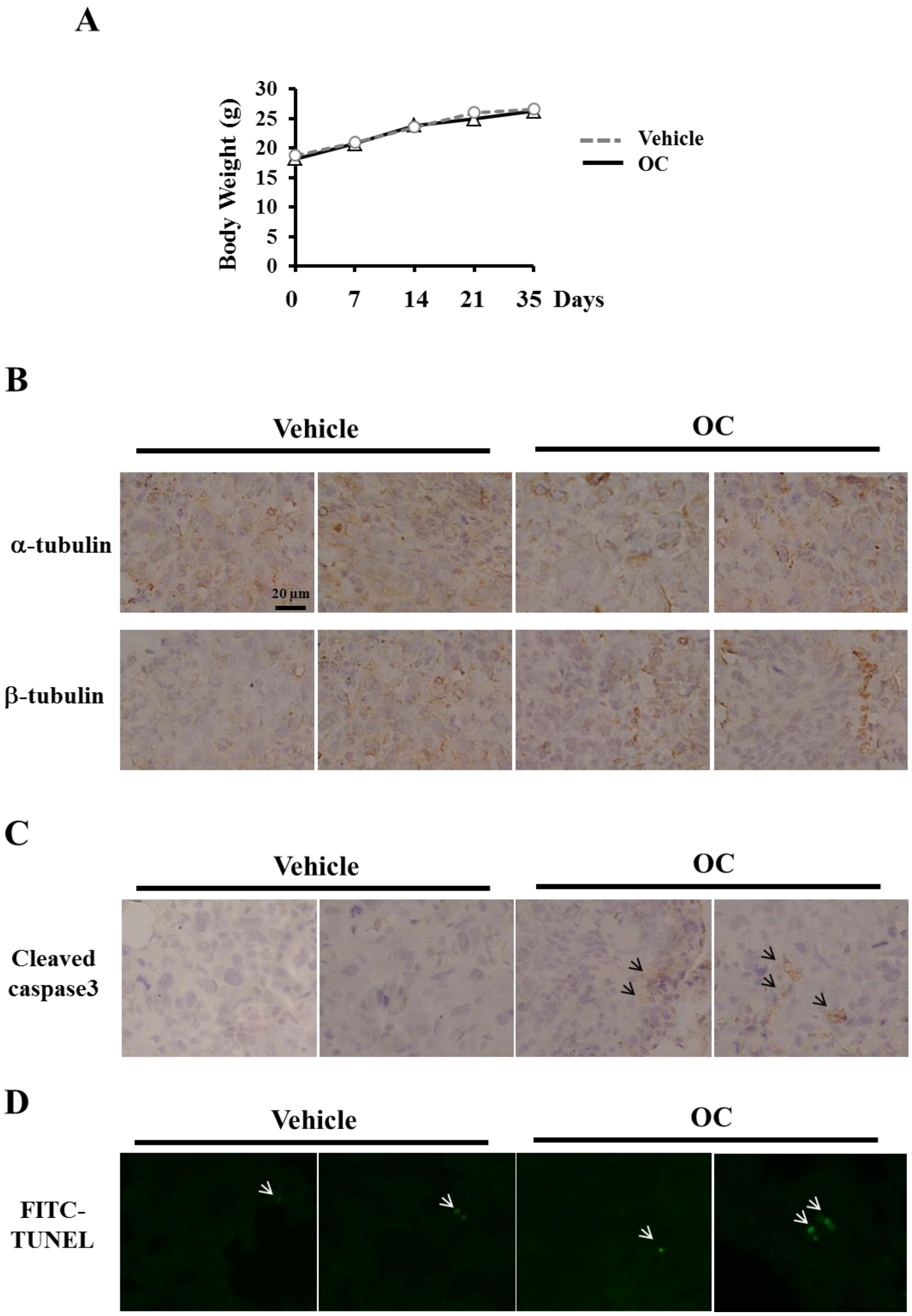

Supplementary Figure 11

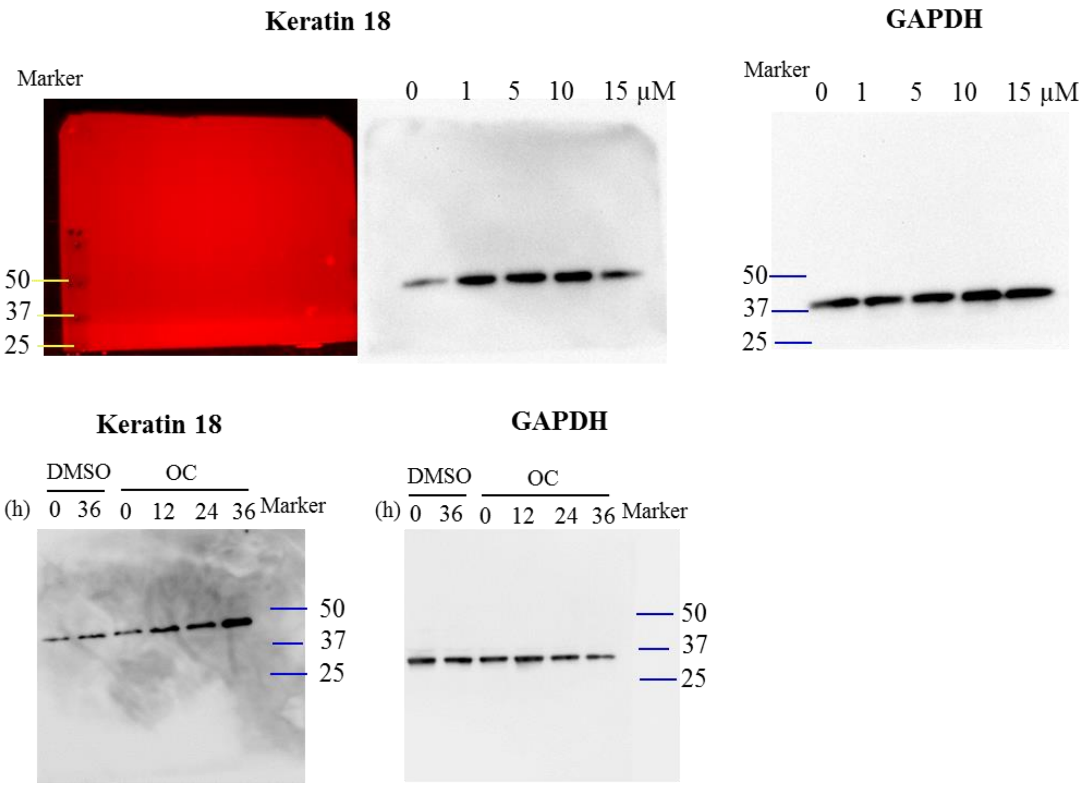

Supplementary figure legends

Supplementary Figure 1.The chemical structure of oblongifolin C (OC).

Supplementary Figure 2. Eca109 cells were scraped, and the migration ability of the cells treated with OC (5 μM), paclitaxel (100 nM) and cisplatin (10 μM) was monitored with an inverted microscope. The images were acquired at 0 and 36 h.

Supplementary Figure 3. OC inhibits cell metastasis in KYSE150 cells. (A) Wound healing assay. KYSE150 cells were scraped, and the migration ability of the cells treated with or without different concentrations of OC was monitored with an inverted microscope. 100 nM paclitaxel was applied as positive control. (B) The cell number from (A) in the wounded regions was counted in each group from three independent experiments. (n=3; *$p<0.05$, **$p<0.01$ vs. vehicle; Dunnett′s test; V: vehicle; P: paclitaxel). (C) Cell viability was accessed by MTT assay in OC treatment for 24 h. (n=8; *$p<0.05$ vs. vehicle; Dunnett′s test).

Supplementary Figure 4. OC inhibits cell migration and invasion in KYSE150 cells. (A) Transwell assay. KYSE150 cells were treated with or without different concentrations of OC for 24 h, subjected to a transwell assay and detected through crystal violet staining. 100 nM paclitaxel was applied as positive control. (B) The cell number in (A) was counted in each group from three independent experiments. (C)

Matrigel invasion assay. KYSE150 cell invasion was analyzed through a matrigel-coated transwell assay. KYSE150 cells were treated with or without different concentrations of OC for 36 h, subjected to a matrigel invasion assay and detected through crystal violet staining. 100 nM paclitaxel was applied as positive control. (D) The cell number in (C) was counted in each group from three independent experiments (n=3; *p<0.05, **p<0.01 vs. vehicle; Dunnett′s test; V: vehicle; P: paclitaxel).

Supplementary Figure 5. OC inhibits cell metastasis in HepG2 cells. (A) The HepG2 cells (treated or not treated with 10 μM OC) that migrated to the wounded regions were photographed. (B) The cell number in (A) was counted in each group from three independent experiments. (C) Transwell assay. HepG2 cells were treated with 10 μM OC for 24 h, detected using a transwell assay and stained with crystal violet. (D) The statistic analysis of cell number in (C) was counted in each group from three independent experiments. (E) Matrigel invasion assay. The HepG2 cell invasion was analyzed through a matrigel-coated transwell assay. The cells were treated with 10 μM OC for 36 h, detected using a matrigel invasion assay and stained with crystal violet. (F) The cell number in (E) was counted in each group from three independent experiments. (n=3; *p<0.05 vs. vehicle; Student′s t-test).

Supplementary Figure 6. Identification of keratin 18 (spot 54) by MALDI-TOF

MS/MS analysis. (A) Silver staining of 2D-gel in DMSO and OC treated HepG2 cells and keratin 18 (spot 54) was highlighted by arrow. (B) The enlarged sections of keratin 18 in the representative silver-stained 2-DE images of HepG2 cells treated with vehicle or OC.

Supplementary Figure 7. OC increases keratin 18 in HepG2 cells. (A) HepG2 cells treated with different concentrations (1, 5 and 10 μM) of OC for 24 h were analyzed by Western blotting for keratin 18. (B) HepG2 cells treated with different concentrations (1, 5 and 10 μM) of OC for 24 h were analyzed by real-time PCR for keratin 18 and GAPDH (n=3; **p<0.01 vs. vehicle; Dunnett′s test). (C) HepG2 cells treated with 10 μM OC over a certain time course were analyzed by real-time PCR for keratin 18 and GAPDH (n=3; *p<0.05, **p<0.01 vs. vehicle; Dunnett′s test).

Supplementary Figure 8. OC does not alter the expression of keratin 8 or keratin 19 in Eca109 cells. Eca109 cells treated with different concentrations (0.1, 0.5, 1, 5 and 10 μM) of OC for 24 h were analyzed by Western blotting for keratin 8, keratin 19 and GAPDH.

Supplementary Figure 9. Keratin 18 knockdown eliminates the OC-induced inhibition of metastasis. (A) Eca109 cells were transfected with keratin 18 siRNA (#1 and #3), and 24 h after transfection, the cells were treated with or without 10 μM OC and analyzed

through a wound healing assay in 24 h. (B) The cell number from (A) in the wounded regions was counted in each group from three independent experiments. (C-D) Transwell and matrigel invasion assay. Eca109 cells were transfected with keratin 18 siRNA (#1 and #3), and 24 h after transfection, the cells were treated with or without 10 μM OC, subjected to a transwell assay (C) or a metrigel invasion assay (D), and detected through crystal violet staining. (E-F) The cell number in (C and D) was counted in each group from three independent experiments. (n=3; *p<0.05 vs. scramble vehicle; [#]p<0.05 vs. scramble OC; Student′s t-test).

Supplementary Figure 10. The effects of OC in a murine experiment. (A) The body weight was measured once a week after OC administration (n=8). (B) Immunohistochemical staining for α-tubulin and β-tubulin in lung sections treated with vehicle or OC. (C) Immunohistochemical staining for cleaved caspase 3 in lung sections treated with vehicle or OC. (D) FITC-TUNEL staining in lung sections treated with vehicle or OC.

Supplementary Figure 11. Full-length blots of Figure 2A and 2B are showed the keratin 18 and GAPDH in Eca109 cells with OC treatment. One representative experiment is showed and the statistical results (Figure 2A and 2B) were acquired from three independent experiments.
